# Neuroinflammation and Parkinson’s Disease—From Neurodegeneration to Therapeutic Opportunities

**DOI:** 10.3390/cells11182908

**Published:** 2022-09-17

**Authors:** Bruna Araújo, Rita Caridade-Silva, Carla Soares-Guedes, Joana Martins-Macedo, Eduardo D. Gomes, Susana Monteiro, Fábio G. Teixeira

**Affiliations:** 1 Life and Health Sciences Research Institute (ICVS), School of Medicine, University of Minho, 4710-057 Braga, Portugal; 2 ICVS/3B’s-PT Government Associate Laboratory, 4710-057/4805-017 Braga/Guimarães, Portugal; 3 Medical and Industrial Biotechnology Laboratory (LABMI), Porto Research, Technology, and Innovation Center (PORTIC), Porto Polytechnic Institute, 4200-375 Porto, Portugal; 4 I3S—Instituto de Investigação e Inovação em Saúde, Universidade do Porto, 4200-135 Porto, Portugal

**Keywords:** Parkinson’s disease, neuroinflammation, inflammatory cells, acute and chronic responses, gut–brain axis

## Abstract

Parkinson’s disease (PD) is the second most prevalent neurodegenerative disorder worldwide. Clinically, it is characterized by a progressive degeneration of dopaminergic neurons (DAn), resulting in severe motor complications. Preclinical and clinical studies have indicated that neuroinflammation can play a role in PD pathophysiology, being associated with its onset and progression. Nevertheless, several key points concerning the neuroinflammatory process in PD remain to be answered. Bearing this in mind, in the present review, we cover the impact of neuroinflammation on PD by exploring the role of inflammatory cells (i.e., microglia and astrocytes) and the interconnections between the brain and the peripheral system. Furthermore, we discuss both the innate and adaptive immune responses regarding PD pathology and explore the gut–brain axis communication and its influence on the progression of the disease.

## 1. Introduction

Neuroinflammation is an immune response to pathogenic stimuli or tissue injury with the primary aim of protecting CNS parenchyma and promoting tissue repair [1]. Actually, acute short-term activation of immune cells is believed to be neuroprotective, inciting local restoration of damaged tissue and toxin/pathogen clearance due to their phagocytic capacity [2]. Nevertheless, the severity of the injury and the persistence of immune reactions may unbalance the neurochemical processes of the central nervous system (CNS). Such events can exacerbate neuronal death, leading to deficits in cell support capacity, blood–brain barrier (BBB) disruption, and, consequently, reaching a chronic and excessive inflammatory response [3,4]. However, the exact cause of such responses remains unclear. In pathological conditions like Parkinson’s disease (PD), an inflammatory cellular response encompasses complex interactions among distinct types of cells [5]. While neuroinflammation is recognized as a key factor in PD, the mechanisms underlying subpopulations of glial cells (i.e., microglia and astrocytes) and their role in disease pathogenesis and progression are still poorly understood. In addition, the abnormal accumulation of alpha-synuclein (α-Syn) is another major hallmark of the disease, which corrupts the normal and endogenous expression of this protein, leading to its pathogenic release and dispersion throughout the brain and into the gastrointestinal tract [6,7,8]. α-Syn is biologically important in synaptic plasticity mechanisms, synaptic vesicles, and neurotransmitter release. α-Syn aggregates can be detected in such biological fluids as plasma or CSF and can be observed in neuronal cells in several brain regions [9]. Moreover, immunoinflammatory cells might also accumulate α-Syn, while astrocytes have been shown to take up the α-Syn molecule and transfer it to other cells [10,11]. On the other hand, microglia are known to collect α-Syn fibrils [12] and contribute to clearance mechanisms, preventing the spreading of the pathology [13]. Notwithstanding the identification of these hallmarks, several questions remain to be answered, especially at the neuroinflammatory level: (1) Which mechanisms/factors trigger neuroinflammation in PD? (2) Which cells or cellular interactions underlie this process? (3) How does neuroinflammation behave during the different stages of PD? (4) Is neuroinflammation a real therapeutic target to attenuate and modify PD progression? Based on such purposes, within the scope of the present review, we discuss the current understanding of inflammatory cells and mechanisms involved in PD initiation and progression. We conclude by summarizing the main therapeutic opportunities concerning the modulation of neuroinflammatory events and opening the window to future perspectives on this topic.

## 2. Microglia in Parkinson’s Disease

### 2.1. Cytokine and Genetic Signature of Microglia in Parkinson’s Disease

Microglia are one of the primary cell types in neuroinflammatory reactions, providing the first line of defense and protection against infection and injury [14]. Under normal conditions, microglia remain in a homeostatic state to survey the brain and spinal cord parenchyma for damage. Nevertheless, they become activated in the presence of infectious agents (e.g., foreign pathogens), prions, pathologically modified CNS proteins, and aggregates, for instance, extracellular space filled with α-Syn aggregates from dying or dead dopaminergic neurons (DAn), apoptotic cells, and immunomodulatory molecules, and sense neuronal activity and neurotransmitter alterations [15,16]. In addition, microglia express pattern recognition receptors (PRRs) at the membrane surface, which can recognize and bind pathogen-associated molecular patterns (PAMPs) and damage-associated molecular patterns (DAMPS), also contributing to their reactive activation [17]. Indeed, it has been shown that microglia proliferate rapidly in response to PRR binding, migrating to the pathological sites and changing their morphology. Still, microglia are also a player in the inflammatory reaction through phagocytosis of cells and debris, releasing a spectrum of proinflammatory mediators [18].

In the context of PD, considering observations from preclinical and clinical studies, the initial microglial response is thought to increase neuronal survival and rescue injured DAn [19]. However, if a sustained and excessive activation of microglia is maintained over time, this has been linked to a deregulated release of proinflammatory cytokines, thereby making microglial cells promoters or contributors to neuropathological and toxicity processes in PD [14]. Indeed, from postmortem analysis, it was found that PD patients presented increased levels of tumor necrosis factor (TNF-α) [20], interleukin-1β (IL-1β) [21], IL-2 [22], IL-4, IL-6 [21], basic fibroblast growth factor (bFGF) [22], and transforming growth factor beta 1 (TGF-β1) [21,23] in the brain. Similar findings were also observed in the cerebrospinal fluid (CSF) of patients, being IL-1β, IL-2, IL-4, TGF-β1, as well as TGF-β2 [24], and TGF-α increasingly detected [23,25,26]. Such release of cytokines forms a unique network in the brain, affecting normal neural and glial function. Microglia themselves are responsible for producing IL-1α, IL-1β, IL-5, IL-6, IL-10, IL-12, TNF-α and TGF-β [27,28]. Some of those cytokines (e.g., TNF-α, IL-1β, and IL6) are being discussed as possible PD biomarkers [29,30,31,32]. In addition, from epidemiological and genetic studies, some PD-associated genes, such as leucine-rich repeat kinase 2 (LRRK2), SNCA (α-Syn), Parkin RBR E3 ubiquitin-protein ligase (Parkin), and PTEN-induced putative kinase 1 (PINK1), have also been linked to neuroinflammatory events [33,34]. Mutations in those genes may affect the normal function of glial cells and may induce microgliosis [15]. With this in mind, Gosselin and colleagues evidenced that microglia and their gene-signature alterations influence (human) brain function [35]. Of note, they showed that microglia present a genetic profile overlapping genes that are up- and downregulated in PD, once microglia can express protein deglycase (DJ-1/PARK7), PINK1, triggering receptor expressed on myeloid cells 2 (TREM2), Parkinsonism-associated deglycase 2 (PARK2), PARK7, SNCA (α-Syn), phospholipase A2 group VI (PLA2G6), ATPase cation-transporting 13A2 (ATP13A2), autophagy-related 5 (ATG5), and glucosylceramidase beta acid (GBA) [36,37]. Modifications in these genes’ activity might impact the ability of microglia to recover neuronal viability and function [38]. In particular, SNCA and LRRK2 genes promote neuroinflammation via activation of microglia and inflammatory signaling pathways through, for instance, nuclear factor kappa B (NF-κB) [15]. Similarly, the GBA gene, which encodes the lysosomal hydrolase glucocerebrosidase (GCase), was found to affect microglial function. Following positron-emission tomography (PET) scans in Lewi body-susceptible brain regions, it was shown that GBA mutations are associated with microglial activation, which consequently fails to protect neurons [39,40]. The work of Keatinge and colleagues supports this role of GBA in neuroinflammation. After creating a GBA knockout in zebrafish, they found that earlier microglial activation had occurred, leading to reduced motor activity, loss of DAn, and ubiquitin inclusions [41]. Nevertheless, the exact molecular mechanism affecting microglia dysfunction remains to be unveiled. Nevertheless, Brunialti et al., suggested that—due to similar features between macrophages and microglia—the same mechanisms (autophagy and lysosomal storage) might be impaired [40]. Additionally, genetic alterations in PD-associated genes, together with α-Syn aggregation and propagation, were also found as key players disrupting other vital microglial processes such as mitochondrial dysfunction [42], autophagy [43,44], and possibly phagocytosis [45,46,47], thereby leading to neuroinflammation and consequently PD development.

### 2.2. Microglia Phenotypic Portrayal in Parkinson’s Disease

**Figure 1:**
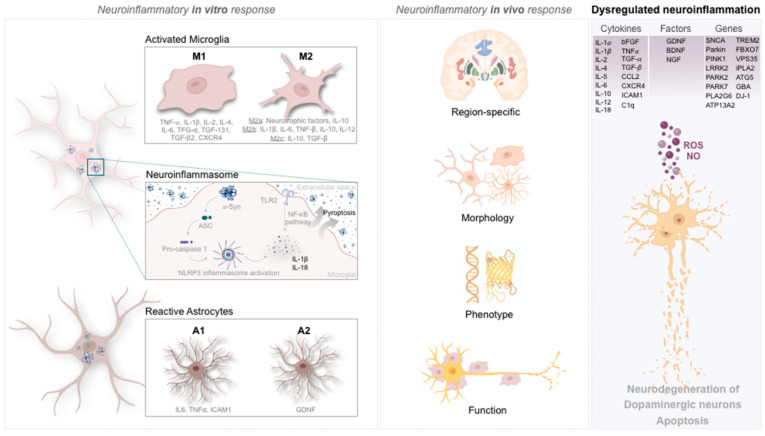
Neuroinflammatory response mediated by inflammatory cells and its impact on dopaminergic neurons in Parkinson’s disease (PD). DAMPs released by dying neurons and CCL2, a proinflammatory mediator, released by astrocytes or by misfolded or aggregated proteins (such as α-syn) trigger the initiation of an inflammatory response. Microglia and astrocytes change their morphology and gene expression and secrete several pro- and anti-inflammatory mediators to restore homeostasis. Canonically, activated microglia can be polarized into M1 (mainly proinflammatory cytokines promoting inflammation) or M2 phenotype (especially anti-inflammatory mediators stimulating repair and regeneration, which can be divided into M2a, M2b, or M2c). Similarly, reactive astrocytes acquire A1 and A2 phenotypes. In PD, the dysregulated cytokine release and the vast abundance of M1 and A1 phenotypes contribute to neurotoxicity and neurodegeneration. M1 phenotype is favorably adopted when DAMPs and CCL2 are released. In astrocytes, the presence of α-syn inclusions leads to the production of proinflammatory cytokines. However, this phenotypic and morphological characterization of inflammatory cells was established according to in vitro studies. NRLP3 (inflammasome) can detect aggregated substances (fibrillar α-Syn in microglia or detected by TLR2 receptors) or IL-1β released by microglia, activating the NF-kB pathway and NLRP3 inflammasome, producing proinflammatory cytokines, such as IL-1β and IL-18, which ultimately leads to pyroptosis. Thereby, in in vivo conditions, microglia and astrocytes exhibit wide heterogeneity. Despite the insufficient techniques to precisely identify the participant cells in inflammatory events in PD, their response is affected by the brain region, morphology, phenotype, and function. The inflammatory response occurring in PD mediated by the release of cytokine growth factors and genetic dysregulation produces reactive oxygen species (ROS) and nitric oxide (NO), leading to neurodegeneration of dopaminergic neurons. (Figure generated using BioRender.com (accessed on 1 June 2022)).

Activated microglia can polarize into several different phenotypes during inflammatory events [48] (Figure 1). Physical and biochemical alterations characterize the different activation phenotypes. The most well-known activation phenotypes are conventionally termed M1 or M2 (Figure 1). However, it is now recognized that this terminology is oversimplified, since microglia can acquire a full spectrum of different activation phenotypes [49]. Concerning the M1 phenotype, this microglial state is correlated with a proinflammatory profile due to the creation of inflammatory environments by releasing cytokines and neurotoxic molecules promoting cytotoxic responses. In contrast, the M2 phenotype (anti-inflammatory) secretes anti-inflammatory mediators and trophic factors that stimulate repair, regeneration, and homeostasis [48]. Both phenotypes play an essential role in normal and pathological conditions [48]. For instance, the proinflammatory state is preferably adopted when dying neurons release DAMPs at the same time that proinflammatory mediators, such as C-C motif chemokine ligand 2 (CCL2), are released from astrocytes [50] in the presence of misfolded or aggregated proteins (e.g., α-Syn), or even signals delivered by Toll-like receptors (TLRs), which generate reactive oxygen species (ROS) and nitric oxide [51]. Proinflammatory microglia upregulate the major histocompatibility complex (MHC) I and II and produce proinflammatory mediators, such as TNF-α, IL-1β, and superoxide [51,52]. In its turn, TNF-α induces apoptosis [53], inhibiting the expression of NF-κB isoforms (e.g., c-Rel) and C-X-C chemokine receptor type 4 (CXCR4), resulting in the degeneration of nigral DAn and development of PD [54,55]. Notably, this was achieved by Parrella and colleagues, who showed that a deficiency in NF-κB/c-Rel leads to the development of early symptoms and progression of PD [56]. Therefore, the proinflammatory factors may worsen and cause widespread damage to neighboring neurons (Figure 1).

Regarding the M2 phenotype, it can be divided into three subgroups. M2a is characterized by the release of neurotrophic factors and IL-10, enabling repair and regeneration. In contrast, M2b expresses proinflammatory cytokines such as IL-1β, IL-6, TNF-β, and the anti-inflammatory IL-10 and IL-12, while M2c might acquire an anti-inflammatory function by releasing IL-10 and TGF-β [36,57] (Figure 1). Equally important are the anti-inflammatory cytokines released that might affect proinflammatory factors in different ways, creating the disparity in phenotypes described, often associated with the development and progression of PD [19]. Therefore, in vivo, microglia cells vary significantly in phenotype, function, and morphology under physiological or pathological conditions [58,59]. For example, their morphology can be shorter, thicker, and less ramified when (re)activated [60].

New emerging research tools, such as epigenetics and single-cell RNA sequencing, have strengthened the idea that the binomial M1/M2 phenotypic classification is in fact a simplistic view initially developed based on in vitro stimulation techniques [59]. Nevertheless, the tools available to identify the participating microglia in vivo are still insufficient [58,59]. In addition to different activation phenotypes, microglia exhibit phenotypic heterogeneity among different brain regions, as described by distinct transcriptome sequencing studies [59,61,62,63,64], suggesting region-specific phenotypes and functional states in a healthy context (Figure 1). On the other hand, in pathological conditions, alterations in microglia morphology synchronize with several functional changes [15]. Indeed, as reviewed by Tan and colleagues, several heterogeneous features, such as microglia abundance, morphomolecular signatures, and homeostatic function, may contribute to distinct responses from microglia upon a pathological stimulus [58]. Furthermore, microglial density is also region-specific, exhibiting different densities throughout the brain. In fact, there are different microglial densities within the basal ganglia—the most affected region in PD—when compared to other brain sites, such as the ventral tegmental area (VTA) [15,61]. Thus, existing variations in microglial density might differentially expose basal ganglia neurons to microglia-derived inflammatory and trophic signaling factors and pathological insults, which, per se, could increase neuronal susceptibility [61]. Most microglia do not reside in the substantia nigra (SN), particularly in PD, where most DAn are located [65]. In accordance, de Biase and colleagues suggested that this fact might contribute to the increased vulnerability of DAn in PD [61] (Figure 1). The activated microglia in nigrostriatal regions of PD patients seem to be neuroprotective in the early stages of the disease, but after a chronic and prolonged activation period, they appear to acquire a neurotoxic role. The communication and cell-to-cell contact between activated microglia and DAn are complex and remain to be understood. Recently, it has been suggested that the degree of microglial activation, inflammatory profile, and intensity depend on microenvironmental circumstances and the neurochemical environment at a particular time throughout the disease progression [66].

Alterations in microglia numbers in different neurological contexts are associated with differential gene expression profiles, which might indicate that these cells can modify their response dynamically. This is an exciting aspect, and a detailed description of these associations might help to determine whether microglia display different activation profiles in distinct diseases [59]. Furthermore, epigenetic studies may be pivotal in providing new insights and unveiling the microglia reactive state in the brain [67]. Beyond these epigenetic studies, the comprehension of microglia anatomy and membrane properties, transcriptome RNA sequencing analysis, cell lineage-tracing tools, high-throughput sorting, high-resolution sequencing technologies, in vivo cell transplantation, and live imaging methods can also contribute to a deeper understanding of microglial biology and their role in pathological conditions [58].

## 3. Astrocytes in Parkinson’s Disease

### 3.1. Cytokine and Genetic Signature of Astrocytes in Parkinson’s Disease

Astrocytes are the most abundant glial cell type in the CNS. They are regulators of synaptic, neuronal, network, and cognitive functions, also being responsible for the metabolic support of neurons [68], BBB integrity [69], and cerebral blood flow [51]. Under pathological conditions and inflammatory reactions, astrocytes can communicate with microglia to amplify the immune response and activate apoptotic mechanisms inducing DAn death [19].

As inflammatory players, astrocytes can be producers of IL-1, IL-5, IL-6, TNF-α, TGF-β, IL-1α, and IL-1β in the brain, while producing granulocyte colony-stimulating factor (G-CSF), granulocyte-macrophage colony-stimulating factor (GM-CSF) and macrophage colony-stimulating factor (M-CSF) in the CSF [27]. Nevertheless, astrocytes are also capable of secreting anti-inflammatory mediators such as glutathione (GSH), ascorbic acid, glial-derived neurotrophic factor (GDNF), brain-derived neurotrophic factor (BDNF), nerve growth factor (NGF) and bFGF [70]. Regarding PD, the accumulation of α-Syn originates in inclusion bodies in astrocytes [71], which might induce alterations in gene expression, proinflammatory cytokine levels (e.g., IL6, TNF-α, intercellular adhesion molecule 1 (ICAM1)) and chemokines [71]. The presence of α-Syn has also been shown to cause severe astrogliosis, disrupting extracellular glutamate homeostasis [72], a crucial feature in astrocytic dynamic function [51,73]. Accordingly, in an A53T PD model, a significant decrease in glutamate and aspartate transporter (GLAST) and glutamate transporter type 1 (GLT1) was observed. Since these are the main transporters of glutamate, their loss of function leads to their accumulation in the synaptic cleft (glutamate excitotoxicity) [72,74,75], contributing to astrocytic and microglial reactivity. Gu et al. demonstrated that the genetic deletion of GLT1 in adult mice leads to a high accumulation of extracellular glutamate, resulting in seizures or even death [72]. In addition to glutamate excitotoxicity, the release of cytokines such as IL1β [76] or TNF-α, as well as the infiltration of leukocytes through the release of vasoactive endothelial growth factor (VEGF) by astrocytes, contribute to BBB permeability [77,78].

Some of the reported overexpressed genes in PD are also expressed in astrocytes, such as PARK2, PARK7, PINK1, LRRK2, SNCA, ATP13A2, PLA2G6, GBA, ATP13A2, F-box protein 7 (FBXO7), and vacuolar protein sorting-associated protein 35 (VPS35) [37,79]. Importantly, astrocytes express a set of genes controlling their activation, lipid metabolism, mitochondrial efficiency, lysosomal function, autophagy, oxidative stress, calcium signaling, glutamate transport, and neurotrophic capacity [80]. Some of these regulatory genes on astrocytes are DJ-1, SNCA, Ca^2+^-independent phospholipases A2 (iPLA2), ATP13A2, PINK1, and Parkin [36,37]. In PD patients, reactive astrocytes increased the expression of lipocalin-2 (LCN2), a molecule secreted by reactive astrocytes under inflammatory conditions [80,81]. Astrocytes release LCN2 to promote their morphological transformation, apoptosis, and migration [81]. Similarly, VPS35, which is more widely expressed in astrocytes than in neurons [79,82], is another component leading to PD risk [83]. This hypothesis is based on VPS35 capacity in regulating transmembrane protein trafficking [83] and microglia morphology [84]. Additionally, decreased levels of ATP13A2 in astrocytes were also found to activate Nod-like receptor protein 3 (NLRP3) inflammasomes, leading to increased production of IL-1β [85].

### 3.2. Astrocytes’ Phenotypic Portrayal in Parkinson’s Disease

Astrocytes become reactive when facing environmental changes or insults, changing their gene expression and morphology (Figure 1). Under such a reactive profile, astrocytes can modify the extracellular matrix, inhibiting axonal regeneration and limiting the spread of damage after injury by creating a glial scar [86]. Transcriptomics analyses indicate that the surrounding microenvironment is crucial to determine reactive astrocyte subpopulations [86]. Studies using modern genetic tools analyzed the cellular, molecular, and functional heterogeneity of astrocytes in the adult brain [15], concluding that, like microglia, astrocytes display region-specific heterogeneity (Figure 1). The region-specific characteristics of astrocytes in the striatum may contribute (as microglia) to a selective neuronal susceptibility in PD [15]. Zamanian et al. analyzed molecular changes using the mRNA profile of quiescent and reactive astrocytes, showing that the nature of the injury or disease contributes to the differentiation of reactive astrocytes into different subtypes [86]. Consequently, it has been suggested that reactive astrocytes play functions according to their transcriptome [86]. Although this assumption did not focus particularly on PD, the complexity of reactive astrogliosis and its gene expression dynamics in injury or disease circumstances may control and interact with the immune response through the release of specific cytokines [86]. In fact, it was concluded that neurotrophic cytokines and growth factor genes are rapidly downregulated in reactive astrocytes whose proinflammatory cytokines and their associated genes are the most persistent.

Similarly to microglia, astrocytes can also acquire different phenotypes. For instance, A1 astrocytes are associated with the gain of neurotoxic function, synapse functions, phagocytosis of altered synapses, and myelin debris [66]. In addition, the secretion of neurotoxic factors promoting the death of neurons and oligodendrocytes is also associated with this astrocytic phenotype [66]. Actually, A1 astrocytes are widely abundant in PD, and microglia interaction appears to activate this phenotype by the secretion of IL-1α, TNF-α, and complement component 1q (C1q) [66]. On the other hand, the A2 astrocytes [86] appear to have a neuroprotective role in the brain, upregulating several neurotrophic factors, such as GDNF, thereby promoting neuronal survival and tissue repair [66] (Figure 1). Therefore, the secretion of inflammatory cytokines, chemokines, growth factors, and the deregulation of gene expression dynamics throughout astrocyte action, along with microglia, might contribute to neurodegeneration events.

## 4. Inflammasome

Neuroinflammatory events occur beyond microglia and astrocytes. For instance, NLRP3, known as an inflammasome, is a receptor located in the cytoplasm of microglia, astrocytes, monocytes, macrophages, neutrophils, and dendritic cells recognizing pathogenic signals [87,88] (Figure 1), and was observed in the peripheral plasma of PD patients [89]. This inflammasome comprises three components: PRR, which works as a sensing molecule, a caspase activating adapter protein (ASC) and an enzymatic component to caspase 1 [88]. Functionally, when facing pathogens, endogenous signals, aggregated substances (fibrillar α-Syn in microglia [90]) or when IL1β is secreted by microglia and sensed by NLRs, the ASC component is triggered [88,91]. Meanwhile, the ASC recruits pro-caspase 1 to be activated [91]. Indeed, the activation of caspase 1 induces posttranscriptional processing of proinflammatory cytokines, such as the IL-1β family and IL-18, leading to the mediation of pyroptosis (cell death triggered by inflammatory signals) [91]. Consequently, these cytokines will activate other cells, amplifying the inflammatory response.

As a result, the persistent fibrillar α-Syn in microglia was reported to activate these cells, triggering the production of IL-1β and consequently activating NLRP3 [90,92]. Its activation produces a proinflammatory cytokine, IL-1β, a molecule augmented in PD animal models and patients [93,94,95]. IL-1β has been identified as a factor essential for the initiation and progression of PD [93]. In addition, α-Syn fibrils can also activate the TLR2 and downstream NF-κB signaling pathway, leading to the synthesis of proinflammatory IL-1β and activation of the NLRP3 inflammasome, beyond the release of other proinflammatory mediators, such as TNF-α [90,92]. Nevertheless, the inflammasome itself also promotes the secretion of inflammatory cytokines IL-1β/18 and induces pyroptosis, exacerbating the dopaminergic cytotoxicity and α-Syn aggregation [96].

Beyond the causes of inflammasome activation already mentioned, mitochondrial dysfunction may also partly explain the activation mechanism of the inflammasome [97]. The altered mitochondrial function, structure, and changes in mitochondrial membrane potential can increase inflammasome activity through microtubules [97,98]. Considering this as a potential therapeutic target, MCC950 is currently a promising therapy. Notably, this selective small-molecule-specific inhibitor of the NLRP3 inflammasome has the power to reduce IL-1β and pyroptosis [99], improving cell viability and survival. Concerning PD, MCC950 was found to inhibit the activation of the NLRP3 inflammasome in the SN and consequently inhibit IL-1β production, improving behavioral impairments, reducing nigrostriatal dopaminergic neuronal degeneration, and disrupting the accumulation of α-Syn inside and outside the nigrostriatal pathway [100,101]. Still, MCC950 exerts neuroprotective effects and high efficacy using nanomolar doses with high target selectivity [100]. For all these reasons, MCC950 or analogues may be a promising therapeutic strategy for future clinical translation to mitigate the progression of PD.

## 5. Acute Versus Chronic Inflammation

### 5.1. What Came First: Neuroinflammation or Dopaminergic Neurodegeneration?

**Figure 2:**
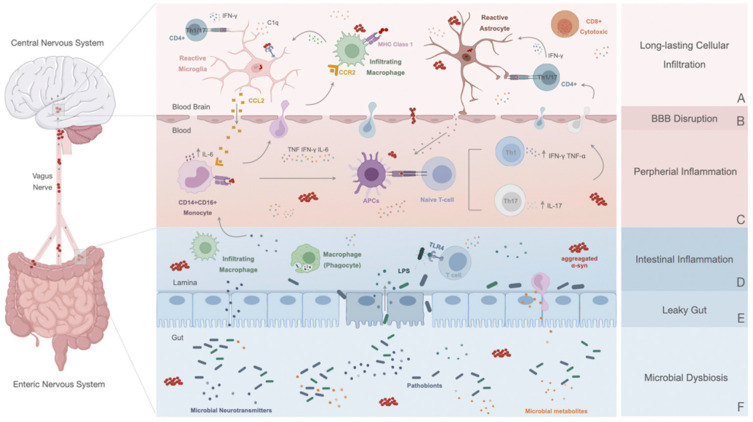
A multisystemic view on Parkinson’s disease neuroinflammation: from CNS gliosis to peripheral immune responses. (**A**) Once neuronal function declines and α-Syn accumulation changes, so will microglia and astrocyte response in PD. The PD brain reaches a chronic inflammatory state characterized by the release of high levels of proinflammatory mediators, continuous degeneration of DAn, and microglia/astrocytic activation. (**B**) With the loss of BBB functionality (associated with microglia and astrocyte reactivity), the exposure of the brain to blood-derived substances occurs, implying a long-lasting infiltration of immune cells, thus reinforcing the local persistent inflammatory response. (**C**) Monocytes could be recruited to the inflamed CNS by activated microglia through the secretion of CCL2. The adaptive arm of the immune system will accompany the immune responses, amplifying a Th1-prone profile (secretion of IFN-γ and TNF-α) and high levels of Th17 cells in the systemic circulation and CNS. Alterations in the status of the ENS, such as abnormal α-Syn accumulation in systemic compartments, intestinal inflammation (**D**), impairments on the intestinal barrier (**E**), and gut microbial dysbiosis (**F**), may favor an inflammatory response in the periphery, promote DAn cell death, and amplify chronic CNS inflammation. (Figure generated using BioRender.com (accessed on 1 June 2022)).

The long-lasting neuroinflammatory response of microglia and the pathological interactions with neighboring, resident glial cells (i.e., astrocytes) and infiltrating immune cells from the periphery (i.e., macrophages and lymphocytes) have been identified as a significant cofactor and contributor of PD progressive neurodegeneration [102] (Figure 2A). Nevertheless, it is not (yet) well understood if microglia under acute or chronic responses solely contribute to PD as facilitators of the disease or if they are a consequence of the disease-initiating process. While some studies have shown that robust immune activation can precede cell death, the opposite is also valid, and cell death per se can induce microgliosis [38]. Thus, understanding how early neuroinflammation occurs in PD is not only a remarkable issue to understand the mechanisms and circuits involved but also to decipher new targeted possibilities to modulate or interfere in the neuroinflammatory process.

Bearing this in mind, extensive analysis of experimental PD models revealed that brain inflammation (i.e., microgliosis) might appear before and independently of DAn death, supporting it as part of the neurodegenerative process. Accordingly, in transgenic animal models with overexpression or mutation (A53T and A30P) of α-Syn, increased numbers of activated microglia and release of proinflammatory molecules were reported, showing that this process can occur before the degeneration of the DAn and the appearance of PD motor symptomatology [103,104]. Similarly, in α-Syn transgenic mice, gene expression pattern analysis in anatomical regions implicated in PD (e.g., SN and brainstem) revealed alterations in the expression of multiple immune-related genes that could arise before the loss of DAn in the SN [105]. Moreover, in a 6-hydroxydopamine (6-OHDA) intrastriatal model, Rodriguez-Pallares and colleagues found an increase in microgliosis and nicotinamide adenine dinucleotide phosphate (NADPH)-derived free radicals 48 h after lesion and before any evidence of SN DAn loss [106]. Also, in rats exposed to rotenone, activated microglia in the striatum and SN were observed before dopaminergic lesions were detected [107]. Using intranigral injection of the immunostimulant lipopolysaccharide (LPS), Castaño and colleagues demonstrated that the nigrostriatal dopaminergic system was susceptible to damage by inflammatory events [108]. In fact, from this study, the authors concluded that the activation of microglia cells occurred in a short time (within two days) and that these could be a cause of progressive DAn death in the long term (to at least 21 days after LPS injection) [108].

Nevertheless, when contrasting preclinical models with clinical data, the evidence supporting microglial influence in neurodegeneration in human patients is still limited. One major drawback is that PD patients are typically diagnosed when a high percentage of DAn degeneration has already occurred, hindering the establishment of a direct connection with microglia inflammation and its potential initiation factors. Despite these considerations, it was recently suggested that a typical midbrain inflammation pattern could occur at the prodromal stage. For instance, data from REM sleep behavioral disorder (RBD) patients—considered at high risk of developing PD (prodromal stage)—has demonstrated that microgliosis could happen years before a possible PD diagnosis [32]. In vivo PET imaging has been used and confirmed this possibility [30,31,32], suggesting that triggering microglial activation is an early and sustained response in PD and is not limited to the areas of significant (DAn) neuronal death. Interestingly, some human studies have also demonstrated an elevation of anti-inflammatory cytokines in the brain and CSF of PD patients, such as TGF-β, identified as an inhibitor of microgliosis [24]. Hence, these findings either suggest that adaptive modifications could emerge in the brain at some point along disease progression, inducing a protective microglia phenotype, or that both pro-and anti-inflammatory microglia might coexist in the PD brain, in which complex changes in microglia phenotypes are likely to maintain and exacerbate the neuropathology. Collectively, these findings propose microglia activation as an early event in PD tissue loss. Even so, if these glial cells can really be responsible for the death of DAn, what are the mechanisms by which they specifically target DAn in an early disease process?

### 5.2. Microglia Activation Preceding the Neurodegenerative Processes: From α-Syn Accumulation to Aging

Studies have suggested that microglia can act as biological sensors and modulate neuronal activity in the brain in the sense that any alteration within DAn, such as changes in neurotransmitter release, ATP production, and synaptic loss [109], could probably initiate a response. For instance, it is known that microglia can express receptors that recognize molecules of neuronal origin, such as neurotransmitters [110]. At the same time, substantial evidence has shown that a potent microglial activation stimulus could be extracellular α-Syn [111]. Regarding this, it has been demonstrated that α-Syn seems to have a chemoattractant ability, inducing direct microglia migration to specific sites in the brain [112]. This coincides with a work reporting that in PD patients, activated microglia closely interact with neurons presenting α-Syn pathological accumulation [113]. Similarly, direct administration of extracellular α-Syn primes the microglia and makes them susceptible to proinflammatory environmental challenge [114], inducing intracellular signaling cascades and modulating inflammatory cytokine production [115] (Figure 2A). However, it should be noted that microglia can also deal with α-Syn during cell-clearing processes [116]. These cells can perceive changes in the structure of endogenous proteins and therefore become activated. Herein, α-Syn might also act as a DAMP, modifying microglia activity and functional capacity, increasing their number and the secretion of proinflammatory molecules (Figure 2A). However, Scheiblich et al. recently discovered that microglia exposed to α-Syn were able to create an ‘‘on demand’’ functional network through the development of F-actin-dependent intercellular connections, which transfer α-Syn from overloaded microglia to neighboring naïve microglia. These neighboring cells, devoid of such pathogenic aggregates, are capable of lowering the individual burden of degradation [12]. This might be an initial mechanism whereby microglia support each other, which could be lost as PD progresses and chronic inflammation takes hold.

To further increase this complexity, aging can be another factor in the equation, as macroglia’s capacity to phagocyte monomeric and oligomeric α-Syn particles declines with age [117]. On the other side of the double-edged sword, it is well known that degenerating neurons may release molecules that spark inflammation, triggering a deleterious feedforward loop [118]. The intrinsic damage in DAn, also called cell-autonomous pathological mechanisms [118], may drive their death and stimulate the secretion of multiple factors by microglia. In fact, DAn can release cellular components that possess intrinsic proinflammatory activity, acting also as DAMPs, such as matrix metalloproteinase-3 (MMP-3), high mobility group box 1 (HMGB1), heat-shock protein 60 (HSP60), ATP, mitochondrial peptides, neuromelanin, and DNA [119]. Therefore, suggesting that cell death could directly trigger microglia reactivity, promoting an immune response within the parkinsonian brain, can be a reality that might endure for several months. This possibility is unreasonable, since it was already shown in a nonhuman primate 1-methyl-4-phenyl-1,2,3,6-tetrahydropyridine (MPTP) PD model that microgliosis was triggered early and persisted for at least 35 months [120].

### 5.3. Astrogliosis Manifestation in Parkinson’s Disease

In addition to DAn, reactive astrocytes and their released proinflammatory mediators can also act on the cognate receptors of microglia, intensifying and leading their reactivity to an overactivated state [121]. Nonetheless, many other potential consequences are being attributed to astrogliosis manifestation. Of note, in PD animal models, astrocytes may play neuroprotective roles in the SN by activating signaling pathways involved in cellular survival/death mechanisms, myelin clearance, and antioxidant defense [122]. Likewise, studies using an MPTP-induced PD model have demonstrated that astrocytes expressing dopamine D2 receptors can modulate deleterious innate immune responses to protect neurons [123]. In this way, to provide energy to neurons that are deprived of nutrients upon insult, these cells might optimize their metabolism to produce lactate, glutamate, and ketone bodies [122], changing morphology and increasing their number to uphold neuron fuel provision and their extracellular homeostasis [122]. Due to this, astrocytes could acquire a senescent profile, which might be crucial for PD development. For example, as shown by Chinta and colleagues, exposure to the herbicide paraquat (PQ) led astrocytes to acquire a proinflammatory senescence-associated phenotype both in vitro and in vivo. This alteration was defined by the release of numerous proinflammatory cytokines, chemokines, growth factors, and proteases [124]. In this way, it is now known that this “conditioned medium” can compromise the viability of DAn. Remarkably, studies have shown that in postmortem PD brain samples, increased astrocytic senescence can also be observed [125], strongly suggesting that this might contribute to the development of sporadic PD.

What is curious and interesting is the significant role that α-Syn can have in this equation. Indeed, it is becoming accepted that this dysfunctional protein might act as an exogenous stimulus to the astrocytic activity [71,126]. In fact, using cultured human astrocytes, it was demonstrated that these cells can uptake α-Syn, subsequently developing inclusion bodies and mediating intercellular transfer to nearby cells [126]. It has also been seen that α-Syn accumulation can lead to the production of proinflammatory cytokines such as IL-1, IL-6, and TNF-α, as well as chemokines such as C-X-C motif ligand 1 (CXCL1) in astrocytic cultures [71] (Figure 2A). Interestingly, reactive astrocytes in the SN of PD patients were highly responsive to inflammatory stimuli and more sensitive to inflammatory reactivation, thus secreting more proinflammatory cytokines [80]. This activation could be, at least in part, through microglial back-and-forth interaction. In previous studies, microgliosis was observed to precede astrogliosis after a lesion [127]. In line with this, Saijo et al. reported that microglia immediately respond to an inflammatory stimulus like LPS or α-Syn exposure [128]. In contrast, astrocytes are later activated by proinflammatory cytokines released by microglia [128]. More recently, Liddelow and colleagues demonstrated that neurotoxic reactive astrocytes can be induced by activated microglia via secretion of IL-1α, TNF, and C1q cytokines [66]. Also, Joshi et al. showed that the propagation of the inflammatory response from microglia to astrocytes might be partially mediated by the release of fragmented mitochondria [129]. Under these circumstances, microglia can also secrete toxic mediators such as glutamate in high concentrations and distinct reactive species [130] that stimulate astrocytic functions, including metabolic routes, leading to overreactivation.

In conclusion, although these microglial–astrocytic (inter)connections may be essential at the onset of PD, by increasing the release of neurotrophic factors, with the progression of the pathological events, and once neuronal function declines and α-Syn accumulation increases, microglia and astrocytes are urged to respond (Figure 2A). This implies that the brain might reach a chronic inflammatory state, which releases high levels of proinflammatory mediators, suffers a continuous degeneration of DAn, and endures microglial–astrocytic activation. In turn, long-lasting immune cell infiltration could also result in an unceasing cycle of inflammation and neurodegeneration (Figure 2A,B).

## 6. Peripheral Response in Parkinson’s Disease

From the immunological point of view, PD is now considered a multisystem disease affecting the whole body [131]. This realization arises from multiple clinical studies and even PD preclinical models, providing robust evidence of an activated innate and adaptive immune response in the CNS and a similar inflammatory reaction occurring throughout the body that may or may not precede DA neuronal loss and clinical motor symptomatology.

### 6.1. Disruption of the Blood–Brain Barrier: A Contributor to PD Immune Dysfunction

It is crucial to underline the evolution of BBB disruption in PD and its diverse impact as the neuropathology develops over a chronic period. Imaging reports have revealed that the BBB of PD patients displays increased leakiness and permeability in different basal ganglia regions, including in postcommissural putamen and SN [132,133], together with cerebral microbleeds (more common in PD patients with dementia) [134] and an overall dysfunction of the BBB transporter system [135]. Additionally, PD patients show vascular remodeling in the putamen and SN pars compacta and abnormal angiogenesis [136]. For instance, in parkinsonian monkeys exposed to MPTP, an increase in VEGF expression was correlated with a boost in the number of blood vessels around degenerating DAn [137], and similar changes were observed in PD patient striata [138]. Conversely, this formation of new vessels might alter BBB susceptibility, enhancing endothelial cells (ECs), pericyte degeneration, and the loss of tight junctions. While the accumulation of neurotoxic material and reduced blood flow can potentiate basal ganglia gliosis, the reverse is also possible. Previous preclinical work in MPTP and 6-OHDA PD mouse models revealed neuroinflammation as an essential contributor to microvascular pathology, especially in the SN and striatum [139,140].

Interestingly, it has been suggested that microglia could be activated before the breakdown of BBB [141], and this trigger could allow the release of TNF-α [142], a cytokine well known for its ability to disrupt barrier function, both in vitro and in vivo [141,143,144,145]. This could stimulate circulating leukocyte infiltration, reinforcing the local inflammatory response (Figure 2A,B). On the other hand, since astrocytes are the major components of the BBB and their end feet are lining the blood vessels, these glial cells could also be potential disturbers of BBB function and integrity in PD (Figure 2A,B) [146]. Depending on the signal they receive from neurons or other cell types and the phase of inflammation, astrocytes might produce VEGF-A [147], a key driver of BBB permeability, which then activates the downstream effector eNOS in ECs and downregulates the expression of occludin and claudin 5 [77]. The cytotoxic milieu generated in PD, composed of cumulative amounts of IL1β, IL6, and TNF-α, could also endorse the activation of ECs and the subsequent release of CC, CCL2, CCL20, CXC, CXCL1, CX3CL1 [148,149,150], ICAM1, vascular cell adhesion molecule 1 (VCAM1) and E-selectin [151], affecting their function and integrity. Hence, initiating an easier infiltration of peripheral immune cells can further potentiate the perceived chronic inflammatory progression. Notably, although there is little information on the contribution of α-Syn to BBB impairment in PD, an in vitro report showed that α-Syn fibril forms cause the functional breakdown of the brain endothelial barrier [152], suggesting that they could affect the intercellular communication between constituent cells of neurovascular units, including pericytes and endothelial cells. Accordingly, it is plausible that BBB leakage might also be mediated by α-Syn exposure and subsequent activation of pericytes through the secretion of high amounts of inflammatory cytokines and MMP-9, even without microglial activation [153].

Thus, with BBB hyperpermeability (Figure 2B) and through the entry of aberrant cells and several neurotoxic derived molecules into the brain parenchyma (i.e., gut-derived neurotoxins), multiple pathways of neurodegeneration can be initiated, mainly targeting DAn, endorsing micro- and astrogliosis, and thus contributing to ongoing neuroinflammation (Figure 2A). While peripheral systemic inflammation is believed to spark and exacerbate the harsh central inflammatory response already emerging in the brain, a correlation between the prodromal stage of PD and peripheral inflammation has recently been established.

### 6.2. Innate Immune Response

Changes in the number and activation profiles of immune cells in PD patients’ blood have been observed (Figure 2C). Indeed, a clinical report from 2018 showed that monocytes and their precursors are upregulated in the peripheral blood of individuals with PD [154]. In general, classical monocytes (CD14^+^/CD16^+^) are enriched in PD patients compared to controls (Figure 2C) and can display pathological hyperreactivity [155]. Specifically, monocytes from patients produce more IL-6 in response to LPS stimulation than healthy controls, which correlates with disease-severity staging [155]. Recent data imply that disease-specific gene expression in peripheral monocytes may connect with distinct disease stages [107]. Through RNA-seq analysis, Schlachetzki and colleagues deciphered a differential monocytic gene expression signature between controls and patients with PD and variations in gene expression dysregulation [107]. The same authors reported a distinctive monocyte signature that breaches PD patients and controls concerning clinical score and disease duration. Interestingly, it was stated that when comparing gene expression in the monocyte cohort versus the human microglia cohort, several genes were expressed at significantly higher levels in monocytes. In fact, genes involved in biological processes such as leukocyte migration and regulation of immune responses were enriched [107], possibly indicating a relation between innate and adaptive immune responses. With this, not only was an early dynamic contribution of monocytes to the disease but their relevance in the clinical manifestation of PD described, which could help to develop predictive models that can decode a temporal course of neuroinflammation.

Conversely, peripheral monocytes could also act as antigen-presenting cells by expressing MHC-I and II, potentiating the ongoing inflammatory response within the basal ganglia (Figure 2C). For instance, monocytic MHC-1, A (HLA-A), MHC-II, DR (HLA-DR), and MHC-II, DQ (HLA-DQ) molecules are assumed to attach to α-Syn, which can posteriorly induce T-cell reactions [156]. In addition, some studies propose that these monocytes are not functionally overactive, but can be stimulated by a “second hit” to exert their abnormal activity. Such “second hits” could be environmental cues or CNS factors eliciting the peripheral immune system and consequent recruitment to lesioned areas [155]. Still, elevated levels of CCL2 in the serum of PD patients, a typical indicator of monocyte recruitment from the bone marrow, have also been linked to this process [157]. Remarkably, activated microglia can also secrete CCL2, which could cross the BBB and recruit monocytes into the inflamed CNS (Figure 2C) [158].

Until now, discriminating monocytes and blood-derived macrophages from endogenous microglia in brain tissue has been highly challenging. Currently, transmembrane protein 119 (TMEM119) is gaining ground as a potential specific microglia marker. Bearing this in mind, in a comparative analysis of five comprehensive datasets of mouse microglia transcriptome, a study has reinforced TMEM119 as a reliable marker for ramified and amoeboid microglia (commonly present in the dysfunctional brain of neurodegenerative diseases, such as PD) [159]. Notably, it was possible to discriminate resident phagocytic microglia from the infiltrating macrophages accumulating in destructive brain lesions, as the latter do not express TMEM119 [159]. Additional data have also shown that only classical blood PD monocytes increase the chemokine surface CCL2 receptor (CCR2) levels, not the resting or activated microglia in the brain [160]. As so, it appears that activating the CCL2–CCR2 axis is necessary to allow monocyte infiltration into the brain [161], which might be critical for their differentiation into blood-derived macrophages within distinct basal ganglia areas and to promote the progression of Parkinson’s neuroinflammatory events (Figure 2C). In a mouse model overexpressing α-Syn, the peripheral recruitment of CCR2+ monocytes was linked to the promotion of neuroinflammation and subsequent DAn death. Furthermore, the posterior genetic deletion of CCR2 was demonstrated to be neuroprotective [161], indicating the deleterious role of infiltrating monocytes in PD. However, in MPTP-treated mice, besides the increased number of circulating and infiltrating monocytes expressing CCR2, it was also observed that these events occurred before the infiltration of T cells, with no effect on neurodegeneration [162].

Accordingly, it has been seen that an increase in TLR4 expression on blood monocytes often occurs in RBD patients [110]. These increases can be related to immune brain activation and indirectly associated with dopaminergic neurotransmission. Thus, this could indicate that a sustained early central and peripheral immune response can occur toward neurodegenerative events (Figure 2C). Nevertheless, despite this increase, monocytes displayed a decreased phagocytic capacity, which is thought to be linked to α-Syn itself [163]. Accordingly, further suggestions are assuming a selective failure or downregulation in the α-Syn uptake process of impaired monocytes, which could represent an important role in PD progression. Altogether, this might indicate that an early compensatory reaction can occur as a way to solve the inflammatory events, which are eventually lost with time, concluding that the course of disease progression is a pertinent aspect to consider when studying immune responses in PD. Although novel evidence has emerged, the precise impact of monocyte infiltration in DAn death still needs to be demonstrated, and further chronic states of infiltration may be required.

### 6.3. Adaptive Immune Response: The Role of T and B Cells in PD

The adaptive arm of the immune system is crucial for detecting foreign antigens and activating different types of lymphocytes, also known as T or B cells [164]. In normal physiological conditions, lymphocytes are not found in the CNS. However, over the last few decades, it has been shown that the peripheral adaptive immune system is also altered in PD, accompanying the CNS-related inflammation and distinct immune responses [165]. PD-related changes in circulating T and B leukocyte populations have been examined, revealing that these cells might be affected by the severity and persistence of the disease, significantly contributing to the chronic inflammation stage of immune reactions in the brain parenchyma (Figure 2C). In the peripheral blood of PD patients, it was possible to observe an altered immune response and a decrease in the overall number of lymphocytes, but not in their frequency [166,167]. For instance, CD4^+^ T-helper and B cells in PD patients are significantly lower than in controls [166,167,168,169]. Interactions between CD4^+^ T-helper and B cells are critical for antibody-mediated immunity, and any changes in these cell populations may compromise immune function in PD. This reduction worsens as the degree of clinical severity increases [167], and a subsequent chronic inflammatory state is attained.

Activated antigen-experienced T cells are predominantly found in the blood of PD patients, indicating an overall shift towards such a phenotype [166]. Interestingly, patients presented increased numbers of memory T cells and a reduced quantity of naïve ones [170], accompanied by a decreased ratio of peripheral CD3^+^ T cells, including CD4^+^. In contrast, numbers of CD8^+^ cytotoxic cells remain unchanged [166,167,168]. As so, it appears that T lymphocytes could display a low CD4^+^:CD8^+^ ratio at a clinical stage, exhibiting a shift from IL-4- to (IFN)-γ-producing cells [167,168]. Whether this decrease in the number of peripheral T cells is due to their migration into the brain remains to be confirmed. Nevertheless, some findings might corroborate this hypothesis. Notably, high levels of activated T cells have been detected in the CSF of patients with PD [171]. Also, it was already reported that an infiltration of CD4^+^ and CD8^+^ T cells (but not B cells) can specifically occur in the SN of the lesioned brain [172]. These cells were near to the blood vessels and neuromelanin-positive DAn [172]. Indeed, postmortem studies have given clear, but static indications of a chronic infiltration of peripheral inflammatory T cells [118], postulating an alteration in BBB permeability, as previously mentioned.

Recently, Kustrimovic and collaborators indicated that the balance among different T-cell phenotypes in the blood of PD patients was biased towards more Th1 response (IFN-γ and TNF-α production) with a reduction in the number of Th2, Th17, and regulatory T cells (Treg) [173]. Indeed, reports have found a change in Treg and a decrease in their ability to suppress the activity of effector T cells in vitro [174,175]. In the brain, this type of lymphocyte might act by upregulating BDNF, GDNF, IL-10, and TGF-β and downregulating proinflammatory cytokines and ROS production, thus suppressing MPTP-induced DAn cell death [176]. A study of Sulzer and colleagues corroborated the capacity of T cells to drive inflammation by uncovering that well-defined α-Syn peptide can act as antigenic epitopes and lead to CD4^+^ and CD8^+^ T cell cytokine responses in PD patients [177]. These observations were based on α-Syn-derived epitopes, particularly epitopes in the phospho-α-Syn (pSer129, a pathological form extant in Lewy bodies), which were found to be recognized primarily by CD4^+^ and CD8^+^ T cells [177]. Further, it was also revealed that T cells can react to α-Syn epitopes derived either from native α-Syn processing, present in the blood, or fibrillated α-Syn. The same study found that around 40% of PD patients displayed immune responses to α-Syn epitopes, reflecting varying disease-progression trends [177]. Various pathogenic alterations have also been found in peripheral blood lymphocytes, such as gaps in the DNA structure, high levels of apoptosis, and Cu/Zn superoxide dismutase activity [178,179].

Supporting this concept are the data obtained from toxin-induced animal models. Of note, considerable information on T-cell infiltration in the SN and striatum (particularly CD4^+^ and CD8^+^) has been acquired using the MPTP model, suggesting T cell-mediated dopaminergic toxicity as a putative contributor to the neurodegenerative process of PD [180,181]. Considering the predominance of infiltrated CD8^+^ over CD4^+^ T cells, when combining the MPTP mouse model with transgenic mice models, Brochard and colleagues observed that CD8*^−/−^* mice (removal of the CD8^+^ T-cell subset) induced a slight loss of dopaminergic cells. In sharp contrast, CD4*^−/−^* mice (where TH deficiency was markedly achieved) mitigated the MPTP injury [172], indicating that the subset of CD4^+^ T cells was the main factor responsible for the cytotoxic effects on DAn, mediating most if not all the harmful activity associated with the adaptive immune response in PD. Indeed, it was shown that mice deficient in T and B lymphocytes remain resistant to MPTP toxicity [180]. As so, recombination activating gene 1 (Rag1) ^−/−^ mice (lacking mature lymphocytes) and T-cell receptor beta chain (Tcrb) ^−/−^ mice (deficient in T cell receptor β) tend to be more resistant to acute MPTP toxicity [180,182]. Research on the overexpression of α-Syn through a recombinant adeno-associated virus demonstrated that infiltration of B and T cells can occur alongside the reactivation of microglia, proposing once again that α-Syn can recall the cells of adaptive immunity and stimulate inflammation [183]. In light of these results, it is possible that T cells could indirectly contribute to PD pathogenesis by influencing the microglial phenotype, causing a shift from an anti-inflammatory to a proinflammatory state or vice versa. In fact, the recognition of α-Syn (presented by MHC-I and -II on microglia) via T cells leads to activation and release of granzymes and proinflammatory cytokines [184,185], significantly contributing to the sustained inflammation and cytotoxic environment. These events are often accompanied by loss of DAn, striatal neurotransmitter depletion, and motor impairments [183]. Likewise, T-lymphocyte infiltration has also been recognized in 6-OHDA models as a time-dependent neuroinflammatory process [127,186,187]. Within this model, an initial decrease of T-reg cells was observed at the peripheral level. Remarkably, this decline corresponded to a phenotypic shift in microglial activation from an anti-inflammatory status (CD206^+^) to a proinflammatory (CD32^+^) one and a consequent decrease in neuronal cells in the SN [186].

Experiments involving the adoptive transfer of T-cell subsets in MPTP-intoxicated mice have hypothesized that not only Th1 but also Th17 cells could contribute to neurodegeneration [176,182]. In a recent study by Sommer and colleagues, high levels of Th17 cells were observed in the blood and postmortem tissue of PD patients [188]. Following this analysis, with human-induced pluripotent stem cell (iPSC)-derived midbrain neurons, the same authors showed that autologous Th17 is implicated in cell death through IL-17A/IL-17R signaling. Nevertheless, these cultures were deficient in glia and other antigen-presenting cells potentially targeting these lymphocytes. Furthermore, α-Syn levels were reduced [188], leading to little knowledge about the role of Th17 antigenic specificity.

Some evidence suggests that human DAn could directly activate CD8^+^ T cells due to the expression of MHC class I molecules, leading to a mediated cytotoxic attack [189]. In addition, a recent work has reported a particular interaction between astrocytes and T cells, assuming that the latter could release neurotoxic factors and promote neuronal degeneration in neurodegenerative disorders like PD [120]. Rostami and colleagues indicated that astrocytes could be highly responsible for T-cell activation in the PD brain [146]. For instance, it has been defended that human astrocytes can accumulate α-Syn, acting as professional antigen-presenting cells and triggering surface expression of costimulatory molecules (CD80, CD86, and CD40) that are essential for activating CD4^+^ T-helper cells in PD [146]. MHC-II-expressing astrocytes are in contact with CD4^+^ T cells in the brain, within and outside the perivasculature (Figure 2A,C). Hence, since astrocytes line blood vessels, they might activate T cells before or during recruitment to the brain parenchyma [146], thereby underlining the importance of these resident cells in establishing T-cell-derived chronic inflammation.

Curiously, mitochondrial antigen presentation (MitAP) can also actively participate in the T-cell cytotoxic response observed in PD. This new conceptual framework has arisen from the studies of Matheoud and colleagues, in which selected proteins from damaged mitochondria were processed for recognition by CD8^+^ T cells [190]. Mutations in the genes PINK1 and Parkin (two significant mitophagy regulators, tightly associated with an early-onset recessive form of PD) strongly contribute to an adaptive immune response (both in the periphery and the brain) by increasing the presentation of mitochondrial antigens and eliciting the establishment of cytotoxic mitochondria-specific CD8^+^ T cells in a PD Pink1-knockout mice model [190]. These data support the role of dysfunctional mitochondrial proteins, providing a new pathophysiological feature where MitAP might affect the immune system and trigger the initiation of autoimmune mechanisms, leading to dopaminergic dysfunction.

Altogether, from the currently available findings, three main conclusions can be established: (a) lymphocyte brain infiltration in PD is not a passive event; (b) neuronal injury and subsequent molecular and cellular changes might regulate site-specific recruitment of T cells into the brain; and (c) T-cell infiltration is most likely associated with neuronal cell death and gliosis in PD, thereby representing a secondary but highly regulated pathogenic event (Figure 2C).

## 7. The Peripheral Enteric Nervous System and the Gut–Brain Axis

Adding to the immune system, the influence of peripheral organs has emerged as an essential niche for studying and understanding the origin and progression of neurodegenerative disorders affecting the brain. In the last few years, a strong connection between the status of the enteric nervous system (ENS) and the function of the CNS has emerged [191]. This has been called the “gut–brain axis,” integrating bidirectional communications between distinct brain areas and the gastrointestinal tract, capable of strongly influencing their activities [191]. Actually, it has been evidenced that gastrointestinal physiology comprising the state of the intestinal epithelial barrier and the activity of intestinal microbes is influenced by signals generated from the brain [192]. Additionally, neurotransmitters, immune signaling molecules, hormones, growth factors, and neuropeptides produced in the intestine can in turn affect the brain [193]. In PD, the significance of gut–brain reciprocal relationships has grown in recent years [194]. It is now well recognized that PD is not only a movement disorder but also a gastrointestinal disease affecting the ENS (Figure 2D–F). In fact, it has become clear that microbial dysbiosis, leaky gut, and intestinal inflammation play a role in the early stages of PD initiation and development, taking a significant role in the prodromal phase of the disease when neurological manifestations are either mild or absent [191]. At this stage, the disease is often documented as an “intestinal syndrome,” where numerous aspects can lead to peripheral α-synucleinopathy and intestinal dysfunction [194]. Chronic constipation is prevalent in PD and can precede motor symptoms by decades [195]. Reports demonstrated that constipation affects 20–80% of PD patients [196,197], and a meta-analysis places the incidence at 50–54% [195], being linked to peripheral inflammation. Emerging evidence suggests that PD patients might be genetically predisposed to gut inflammation [198]. As a matter of fact, in various illnesses associated with gastrointestinal tract disturbances, such as inflammatory bowel disease (IBD) and ulcerative colitis, several PD-risk genes, such as LRRK2 [199] and caspase recruitment domain-containing protein 15 (CARD15) [200], are shared. As so, it has been seen that for different IBDs, particularly Crohn’s disease, the risk of developing PD in certain populations increases [201].

### 7.1. Altered Gut in Parkinson’s Disease: A Promotor or a Consequence of the Inflammatory State?

It can be hypothesized that gut-derived inflammation plays an important pathogenic role in the disorder, as there are recent indications that it can contribute to PD initiation and progression (Figure 2D). In fact, Devos et al. found increased levels of proinflammatory cytokines (e.g., TNF, IFN-γ, IL-6, and IL-1β) and expression of glial activation markers (GFAP and Sox-10) in the ascending colon of PD patients in comparison to healthy individuals [198]. Remarkably, these cytokines are thought to be higher in number in early PD stages and consequently decline with time [198], which indicates that gut inflammation could be a primary event in the pathogenic processes of PD (Figure 2D). Given these observations of intestinal dysfunction and inflammation in PD individuals, it is also logical to dissect possible changes in the gut microbiome.

Besides being very important in overall gut function (exclusively responsible for several metabolic processes, such as the production of short-chain fatty acids and (SCFAs) vitamins and amino acid synthesis (AAs) [191]), the intestinal microbiota also shapes the immune system of individuals by controlling the differentiation and function of immune cells in the intestine and brain [202,203]. Alterations in gut microbiota such as dysbiosis can disturb brain neurochemistry (altered levels of neurotransmitters, receptors, and neurotrophic factors [204]), behavior [204,205], and the regulation of synaptic plasticity levels [204]. Overall, the gut-microbiome composition is altered in PD patients compared to healthy controls, and these findings have been replicated in multiple studies across various geographies [131,206].

In line with this, the assessment of altered gut microbiota in fecal samples of PD patients was determined, and distinctive stages of disease progression displayed different microbial compositions [207,208,209]. More recently, a cohort study was conducted to specify gut microbial alterations in PD using microbiome-wide association [210]. This study revealed that microbiota composition in PD appears to be deficient in microbes associated with an “anti-inflammatory” environment and normal metabolic activities, but enriched in pathobionts that stimulate inflammation (Figure 2E,F). A considerable increase in carbohydrate-metabolizing bacteria (Lactobacillus and Bifidobacterium) and opportunistic LPS-secreting pathogens was also observed, which may induce damage to host tissue. Conversely, with the decreased abundance of SCFA-producing bacteria [210], the gut may become a site predisposed to inflammation (Figure 2E,F). Thus, changing the plasma cytokine profile [206], pathological consequences of intestinal dysbiosis comprise immune activation and the involvement of TLRs [211]. In a study conducted by Perez-Pardo et al., it was observed that TLR4 responded to bacterial LPS [212], thus suggesting a role in mediating microbiota inflammatory processes. Stool-sample analysis showed higher TLR4 gut levels, T cells, and cytokines in PD patients [212]. Following this, it was demonstrated that intestinal and brain inflammation was less extensive in TLR4-knockout mice than in wild-type ones [165].

Additionally, recent reports have described that pathogenic gut microbiota, through the systemic release of various metabolites (such as LPS), can increase intestinal epithelium permeability and subsequent damage to barrier function [213]. These intestinal barrier impairments result in the leakage of microbiota and their metabolites from the gut into the circulation, activating inflammatory cascades that can induce systemic inflammation (Figure 2C–F), as reviewed by Rani and colleagues [213]. The elevated exposure of microbiota-produced substances into the CNS disrupts BBB permeability, triggering neuroinflammation and DAn degeneration in the SN [214]. A study conducted by Sampson and colleagues described that those alterations in the intestinal microbiota could also endorse motor symptomology in a mouse model of the disease [215]. The same authors performed fecal transplantation from PD patients to healthy mice and observed significant motor-function deterioration [215]. Remarkably, they also identified specific microbiota metabolites present in the feces of patients that are sufficient to promote PD symptoms [215]. It is also known that in mouse models of gut injury, the gut microbiota can penetrate injured areas and induce macrophages to migrate to the damaged sites [216].

### 7.2. Gut–Brain Communication: α-Syn Spreading and Novel Initial Sites of Inflammation

Another intestinal feature of PD that has been widely reported is the presence of α-Syn enteric abnormalities in the colon, neurons of the ENS, and within the vagus nerve itself [10,217]. This protein is expressed as a standard component of the ENS, and it can be detected in intestinal tissue in a large percentage of neurologically intact humans [218,219]. Nonetheless, it is noticed more frequently and at higher levels in the intestines of PD patients than in age-matched healthy controls [217,219]. Therefore, if they first appear in the gut, how do α-synucleinopathies extend to the CNS? In 2003, Braak and colleagues introduced the hypothesis that PD pathology and protein accumulation may initially be triggered in the gastrointestinal tract and then spread through the dorsal motor nucleus of the vagus (DMV) to the caudal brainstem and eventually to the SN, like a prion-like disease [220]. This idea was sustained by the pathophysiological presence of α-Syn protein inclusions in the ENS and the glossopharyngeal and vagal nerves in the early stages of PD [217]. Furthermore, it has been postulated that the vagus nerve could be a critical pathway of retrograde transport of α-Syn between the ENS and the brain. In a recent study using an α-Syn mouse model, researchers found that truncal vagotomy preceding α-Syn deficiency prevented gut-to-brain spread of α-synucleinopathy, the associated neurodegeneration occurring in the SN and striatum, and motor and nonmotor deficiencies [221]. Similarly, cervical vagotomy has also been shown to effectively prevent the formation of α-Syn aggregates in mice after inoculation with α-Syn preformed fibrils (PFF) into the gastric wall [222].

Additionally, it has been suggested that in a prion-like manner, the cell-to-cell transmission of α-Syn through the vagal nerve may require the immune system’s involvement [213]. In a vicious circle, the cell-to-cell transmission of misfolded α-Syn aggregates from the ENS into the brain may further contribute to the sustained activation of local macrophages [223] and disease progression. As such, this gut–brain axis disruption and α-Syn accumulation have been seen in many animal models of PD, confirming the involvement of the vagus nerve (Figure 2). For instance, in PD rats, both vagal efferent axons and terminals express α-Syn [224]. Similarly, while the injection of α-Syn fibrils into the intestinal tissue of healthy rodents is sufficient to induce pathology in the vagus nerve [225], the installation of rotenone into the stomach exhibits an advanced accumulation of α-Syn in the ENS, the vagus nerve and subsequently in the brain stem [226]. In accordance, various in vivo studies have shown that gut dysbiosis may induce the accumulation of α-Syn in the ENS with pathological consequences. For example, in mice overexpressing α-Syn, exposure to microbial metabolites derived from patients originated neuroinflammation and motor symptoms [227]. Interestingly, a transgenic α-Syn PD model showed that the existence of gut microbiota crucially influences motor deficits and pathological disturbances [215].

Nevertheless, in the opposite direction, the overexpression of α-Syn in the SN has led to ENS changes and altered microbiota [228]. A supplementary mechanism that could promote α-Syn accumulation and aggregation is the synergistic neurotoxic effect of chronic (peripheral) LPS [229,230]. Data have demonstrated that this inflammatory metabolite released by intestinal microbiota could bind to α-Syn and start its fibrilization in the gut, propagating via the vagal nerve into the brain [215]. A transgenic mouse (overexpressing human A53T mutation) exposed to LPS revealed an increase in neuroinflammation, protein aggregation, DAn loss, and Lewy body (LBs) formation in the nigrostriatal neurons compared to wild-type animals [230]. Moreover, intraperitoneal injections of LPS (before α-Syn intravenous administration) were found to direct α-Syn internalization via inflammatory monocytes [231]. As cited, these cells can infiltrate the brain, hinting that the peripheral activated monocytes could also act as a Trojan horse in PD [231], promoting the entrance of peripheral (modified) α-Syn to the CNS. Curiously, the potential triggering of enteric glial cells (EGC) inside the gastrointestinal tract of PD patients, perceived in the early phases of the disorder, might amplify the intestinal barrier’s impairment and facilitate the spread of pathological α-Syn within the ENS [194].

Beyond the gut–brain interaction, the olfactory bulb has been recently considered a possible initial site for α-Syn spreading, namely due to (1) the modification of α-Syn found in the olfactory bulb neurons, which might predict the brain pathology in the CNS [232]; (2) the notable neuronal loss in regions connected to olfactory structures that are observed in about 90% of PD patients; (3) the smell loss that appears years before any motor symptoms [233]; and 4) the total volume of glomeruli in the olfactory bulbs that are diminished in 50% of patients [234], in which it is believed that the olfactory bulb could be the starting or the intermediate point of the inflammatory response. So far, only one study has demonstrated microgliosis in the olfactory bulb in PD patients [235]. However, in a rat model, intravenous LPS administration provoked a robust inflammatory response in the olfactory bulb, increasing peripheral immune cells and levels of common proinflammatory cytokines [236].

Multiple observations of human and animal models have linked the abnormal immune system actions and PD pathogenesis to (i) systemic inflammation, (ii) infiltration of peripheral immune cells in the CNS, (iii) hyperreactive circulating immune cells, (iv) gut microbial dysbiosis, and (v) abnormal α-Syn accumulation in systemic compartments outside the CNS (Figure 2). Altogether, the connection between CNS-specific immune mechanisms and the peripheral innate and adaptive immune system in the initiation and progression of PD has become far too substantial for modern PD research to ignore.

## 8. Therapeutic Approaches to Target Neuroinflammation in PD

Several drugs can be highlighted among the therapies potentially treating or delaying PD, as they have presented positive effects in preclinical and clinical studies. Nevertheless, the lack of effective treatments for PD has prompted the search for novel therapeutic options. Interestingly, neuroinflammation is emerging as one of the features that can be targeted in this pathology. This section highlights novel pharmacological approaches for treating PD from a neuroinflammatory perspective.

### 8.1. Immunotherapy for Alpha-Synuclein (α-Syn) Aggregation

As previously described, toxic α-Syn forms affect neuronal function and activate several immune pathways and cells. These harmful outcomes might occur due to failure in the clearance of α-Syn aggregates [237]. Considering that PD patients present low levels of α-Syn antibodies, the clearance mechanisms seem to be damaged. Therefore, an immunotherapeutic approach would allow the blocking of the formation of extracellular α-Syn assemblies to block oligomerization, fibrillization, and/or aggregation of α-Syn, thereby avoiding cell-to-cell propagation [165]. Antibodies produced by animals’ immune systems against α-Syn (passive immunization) or the administration of antigens to induce the production of antibodies (active immunization) have stood out as therapeutic approaches to target neuroinflammatory impairments in PD [238,239] (see review [240]). Indeed, the first vaccine produced—PD01A—was compelling and successfully applied, causing the production of antibodies against aggregated α-Syn, reduced deposition, and improved memory and motor defects in mouse models [241]. In PD patients, PD01A was demonstrated to be safe and well tolerated for extended periods in a phase I clinical trial [241], but more tests are needed to ensure its efficacy (Table 1).

### 8.2. Vagotomy and Appendectomy

Due to the gut–brain interaction and consequent α-Syn aggregation from the gastrointestinal tract to the lower brain stem, vagotomy and appendectomy appear as possible treatments to reduce the risk of developing PD [2,221] Some cohort studies presented evidence for a potential protective effect of truncal, but not selective vagotomy, against PD development [252,253]. In fact, individuals who underwent truncal vagotomy had a 15% lower risk of developing PD, as stated by Svensson and colleagues [254]. Therefore, these reports support that the vagus nerve participates in the transmission of pathological α-Syn within the gut-to-brain axis (Figure 2), by which vagotomy may delay rather than eliminate the risk for PD. Additionally, in another cohort of patients, it was shown that the appendix might have a crucial role in PD through inflammatory and microbiota alterations because the appendix is a rich and lifelong source of misfolded αSyn, which means that the early removal of this organ relates to a diminished risk of PD development [255]. Altogether, a better understanding of the interaction between the gut–brain axis, the gut microbiota, and PD has the potential to lead to new diagnostic and therapeutic strategies.

### 8.3. Nonsteroidal Anti-Inflammatory Drugs

Anti-inflammatory agents such as nonsteroidal anti-inflammatory drugs (NSAIDs) may suspend the progression of the inflammatory response, minimizing the risk of exacerbated feedback in PD. One of the best NSAID candidates is ibuprofen. It plays an anti-inflammatory role by nonselectively inhibiting the activity of cyclooxygenase (COX), an enzyme that catalyzes the synthesis of prostaglandins and is upregulated in dopaminergic neurons of PD patients [256]. Furthermore, ibuprofen seems to have antioxidant properties that promote an alternative mechanism for neuroprotection independently of COX inhibition [257,258]. Thus, ibuprofen mitigated dopaminergic neurodegeneration by preventing the formation of oxidative species in animal model studies [256]. However, this pharmacological approach still lacks clinical validation in a PD context [259].

Beyond ibuprofen, other NSAIDs, such as aspirin and celecoxib, showed protective effects in PD pathology [260]. Aspirin, also an inhibitor of COX, affects neuroinflammatory processes and neuronal degeneration, preventing striatal dopamine depletion [261]. Therefore, further exploring NSAIDs as a therapeutic strategy could potentiate the modulation of neuroinflammatory events associated with PD.

### 8.4. Food-Based Therapies and Physical Exercise

Dietary therapies seem to reduce gut permeability, oxidative stress, and intestinal inflammation and ultimately balance the microbiota dysbiosis [262]. Targeting the gut–brain axis with the use of probiotics (i.e., *Lactobacillus*, *Bifidobacterium*), prebiotics (i.e., inulin, galactooligosaccharides, fructooligosaccharides, short-chain fatty acids), and symbiotic agents (combination of probiotics with prebiotics) that affect gut-microbiome homeostasis could be a promising tool to consider (see review [263]). On the other hand, polyunsaturated fatty acids in high quantities have anti-inflammatory effects and may reduce NLRP3 inflammasome activators, namely, α-Syn aggregation and mitochondrial dysfunction [264,265,266].

A healthy diet based on fresh vegetables, fresh fruits, nuts, seeds, nonfried fish, olive oil, wine, coconut oil, fresh herbs, spices (Mediterranean diet), [264], and flavonoid-rich food (i.e., tea, apples, oranges, red wine) could be beneficial and protect against the development and/or progression of PD [267]. Additionally, caffeine consumption has been linked to a reduced risk of PD (see review [268].

Beyond a healthy diet, physical activity improves both motor and nonmotor PD symptoms, slowing disease progression, reducing neuroinflammation, delaying DAn loss, and increasing synaptic connectivity [269,270]. Aerobic exercise in PD patients was found to increase BDNF levels in the serum, as well as decrease the levels of inflammatory markers, such as VCAM and TNFα, leading to a decrease in microglial activation and oxidative stress and an increase in dopamine and neuroplasticity levels [269,270,271,272]. Physical exercise also decreases the effect of PD molecular hallmarks such as α-Syn accumulation and mitochondrial dysfunction, rescuing DAn loss in rodent models of PD, improving antioxidative capacity, and reducing levels of the proinflammatory cytokine IL-1β [271,272]. In summary, exercise exerts neuroprotective effects and mitigates inflammation in PD.

It is worth highlighting that various clinical trials have already been performed aiming to recover the debilitant conditions of patients suffering from PD, most of them focusing on reducing/inhibiting extracellular α-syn and enhancing autophagy levels or stimulating molecular chaperones for the lysosomal enzyme GCase. Recently, new clinical trials have been developed employing PET ligands, such as GE180 (NCT03702816) and [18F]DPA-714 (NCT03457493), which bind to the mitochondrial translocator protein (TSPO), whose presence can be associated with inflammation. TSPO is in the mitochondria of activated microglia. Thus, regional and global inflammation can be analyzed in living patients in a noninvasive manner through PET imaging. The main goal of these studies is to determine whether PD patients present more neuroinflammation than healthy patients. Until now, no publicly available results exist, but these studies may provide a better understanding of inflammation in PD (Table 2).

Beyond the therapies explored in this section, other classes of pharmacotherapies mentioned in Table 3 may tackle different targets producing beneficial impact through the distinct mechanism of action. Those drugs seem to be equally effective, even if clinical tests and analysis are indispensable.

## 9. Gaps in the Literature and Future Perspectives

Neuroinflammation is a hallmark of PD, and its mechanisms are well studied and explained in the literature. However, despite the pharmacological treatments under clinical trials, there are still no effective candidates to target PD-related inflammatory events. The inflammatory processes and deregulations can occur in parallel with DAn degeneration and α-Syn aggregation. Due to the molecules that can trigger inflammatory events, neuroinflammation is not seen as a cause but a consequence of CNS alterations.

Keeping in mind the current clinical tools to diagnose PD and the remaining unknown cause, deeper elucidation of the alterations caused and affected by neuroinflammation in the CNS needs to be addressed. For instance, proteomic, transcriptomic, and epigenomic studies in inflammatory cells should be considered. In vitro studies and in vivo data from models that strongly mimic the conditions in human patients could be used to determine the exact moment or the cause that turns acute inflammation into the chronic and peripheral response observed. By doing so, suitable and effective therapies to stop the vicious circle of inflammatory responses will probably emerge in the near future. In addition, healthy dietary routines and improvements in lifestyle could also work as preventive components to avoid gastrointestinal and immune dysfunction. However, the neuroscience community has scarce specific knowledge regarding the effectiveness of prebiotics and symbiotics in PD patients. Therefore, new protocols must be developed to examine these issues both in vitro and in vivo, as well as strategies to modulate them. By doing this, it will be possible to understand if inflammation, the gut–brain axis, and the cells involved in it play a role in PD initiation and progression and may be used as real therapeutic targets for PD.

## Figures and Tables

**Table 1:** Treatments for neuroinflammation in Parkinson’s disease animal models.

Treatment	Target	Species Tested	Results	Reference
Animal Models-Active Immunization				
Vaccination of human aSyn	α-Syn	Transgenic mice human α-Syn	Promoted degradation of human α-Syn aggregates Ameliorated the loss of synaptophysin-immunoreactive nerve terminals	[242]
PSDC (peptide-sensitized dendritic cells)	Vaccine based on dendritic cells sensitized with α-Syn	Transgenic mice that expressed the human disease-associated A53T mutation of α-Syn with bone marrow-derived dendritic cells	Restored mobility Lower levels of the proinflammatory cytokine IL1-α	[243]
DNA vaccination	Induced overexpression of growth factors	C57BL/6 mice	Reduced cell death neurons in the SN and cyclooxygenase 2 expression Better performance in motor functions	[244]
Animal models—passive immunization				
9E.4	C-terminus of α-Syn	Transgenic mice PDGF-hu-wt-α-Syn	Improved motor and cognitive performance Reduce the oligomerized α-Syn aggregates Ameliorated neuropathological alterations	[245]
AB274	C-terminus of α-Syn	Transgenic mice PDGF-hu-wt-α-Syn	Reduction of inflammatory cytokines (TNFα and IL-6) Extracellular clearance of α-Syn Prevented α-syn spreading from neurons to astroglia Ameliorated neurodegeneration Improved motor and cognitive performance	[246]
1H7, 5C1	C-terminus of α-Syn	Transgenic mice PDGF-hu-wt-α-Syn	Reduced accumulation of α-Syn Prevented loss of TH fibrils in striatum Improved motor and memory deficits	[247]
5D12	C-terminus of α-Syn	Transgenic mice PDGF-hu-wt-α-Syn	Reduced α-Syn clearance in the cortex and striatum	[247]
Ab47	Protofibrils of α-Syn	Transgenic mice expressing the human pathologic A30P variant of α-syn under a Thy1 promoter (Thy-1)-h[A30P] α-Syn transgenic mice)	Reduced levels of toxic α-Syn protofibrils Improved motor symptoms	[248]
Syn303	N-terminal of α-Syn	C57BL6 mice	Reduced α-Syn aggregates in striatum Less dopaminergic neuron loss in the SN Decreased transneuronal α-Syn transmission Decreased microglia activation Improved motor performance	[249]
AB1	N-terminal of α-Syn	Harlan rats injected with AAV-α-Syn-AB1	Reduced TH-positive neurons and neuroinflammation. Attenuated α-Syn accumulation in SN Decreased microglia activation Prevented dopaminergic cell loss Improved behavioral deficits	[250]
AB2	Central region of α-Syn	Harlan rats injected with AAV-α-Syn-AB2	Reduced neuroinflammation Ameliorated behavioral deficits	[250]
AFF1	C-terminus of α-Syn	Transgenic mice PDGF-hu-wt- α-Syn Or Transgenic mice mThy1-α-Syn	Reduced the accumulation of α-Syn oligomers Clearance of α-Syn Increased anti-inflammatory cytokines expression Improved motor behavioral and memory deficits	[251]

**Table 2:** Pharmacotherapies tested in clinical trials with Parkinson’s disease patients (ClinicalTrials.gov, accessed on 1 May 2022).

Treatment	Target	Criteria	Phase	ClinicalTrials.gov Identifier	Description	Company/Class	Reference
Clinical trials for reducing extracellular α-Syn							
PD01A	Oligomeric α-Syn	45 and 65 years old with early-stage idiopathic Parkinson’s disease on stable medication	II (To begin)	NCT 01568099	Active vaccine to α-Syn composed by amino acid peptide	AFFirRIS	[241]
PRX002	C-terminus of α-Syn	Patients with early PD who are untreated or treated with MAO-B	II (Ongoing)	NCT03100149	Monoclonal antibody	Prothena	[273]
BIIB054	N-terminal of α-Syn	40–80 years	II (Ongoing)	NCT03318523	Human-derived α-Syn antibody	Biogen	[274]
MEDI1341	α-Syn	Healthy volunteers aged 18 to 65 years	II (Completed)	NCT03272165	Monoclonal antibody	AstraZeneca	[275]
805 BAN	Oligomeric/protofibrillar α-Syn	Patients with idiopathic, mild to moderate Parkinson’s	I (Ongoing)	NCT04127695	Humanized monoclonal antibody targeting α-Syn	Abbvie/Bioarctic	
UB-312	C-terminus of α-Syn	Healthy participants and participants with PD	I (Ongoing)	NCT04075318	UBITh-enhanced synthetic peptide-based vaccine	United neuroscience	[276]
Clinical trial for GCASE stimulation							
Ambroxol	β-glucocerebrosidase pathway/expression	40–80 years	II (Completed)	NCT02941822	Mucolytic compound that acts as a molecular chaperone for the lysosomal enzyme glucocerebrosidase (GCase)	Cure PD	[277]
Clinical trial for inhibiting αSyn aggregation							
NPT200-11	α-Syn	18–55 years	I (Completed)	NCT02606682	Small-molecule inhibitor of α-Syn misfolding and aggregation	Celerion/NeuroPore	
Clinical trials for enhancing autophagy							
Nilotinib	Abl tyrosine kinase inhibitor	40–79 years with idiopathic PD	II (Completed)	NCT03205488	Abl tyrosine kinase inhibitor	Michael J. Fox Foundation for Parkinson’s Research	[278]
K0706	Abl tyrosine kinase inhibitor	More than 50 years early PD not receiving dopaminergic therapy	II (Ongoing)	NCT03655236	Suppressor of an enzyme called Abl tyrosine kinase	Sun Pharma Advanced Research Company Limited	[279]
Other recent clinical trials							
GE180	Mitochondrial translocator protein (TSPO)	55–90 years	Ongoing	NCT03702816	PET ligand	The Cleveland Clinic	
[18F]DPA-714	Mitochondrial translocator protein (TSPO)	30 years and older	Ongoing	NCT03457493	PET tracer	University of Alabama at Birmingham	
Leukine	Granulocyte-macrophage colony stimulating factor receptor	35–85 years	I (Ongoing)	NCT03790670	Human recombinant granulocyte macrophage colony-stimulating factor expressed in yeast	University of Nebraska	[280]

**Table 3:** Potential pharmacotherapies suitable to pursue to treat Parkinson’s disease.

Treatment	Target	Description	Class	Reference
Potential Therapies for PD				
Ibuprofen	Inhibitor of COX	Prevents microglia proliferation through modulation of cell cycle progression and apoptosis Reduces neurodegeneration-related glial inflammation	NSAIDs	[259,281]
Aspirin	Inhibitor of COX	Ameliorates the inflammation in the brain Prevents dopamine depletion in the striatum	NSAIDs	[261]
Salicyclic acid	Metabolite of aspirin	Reduces behavioral impairments Reduces dopamine depletion Inhibits glia inflammation in dopaminergic neurons	NSAIDs	[282]
MCC950	Walker B motif within the NLRP3 NACHT domain	Inhibits the activation of NLRP3 inflammasome in SN and consequently inhibits IL-1β production Improves behavioral impairments Reduces nigrostriatal dopaminergic neuronal degeneration Disrupts the accumulation of α-Syn	Inflammasome inhibitor	[99,100,101]
Inosine monophosphate dehydrogenase (IMDH) inhibitors	Intracellular signaling pathways	Suppress propagation of peripheral αSyn aggregation in the gastrointestinal tract Suppresses T cells response	Immunosuppressors	[283,284]
Minocycline		Anti-inflammatory and neuroprotective properties in PD Reduces the progression and severity of disease Mitigates neuroinflammation in the adult and aged brain Modulates cytokine-associated changes in behavior	Antibiotic	[285,286]
Dexamethasone	Cytoplasmic glucocorticoid receptor	Less dopamine depletion and less decrease in the number of dopaminergic cells in the SN Decreases the inflammatory reaction: glial activation, lymphocytic infiltration Exerts a neuroprotective effect May be an inhibitor of inflammatory process	Glucocorticoid	[287]
Naloxone	μ-Opioid receptors	Decreases microglia activation and increases the level of proinflammatory cytokines Improves cognitive dysfunction	Opioid	[288]
